# Beyond redundancy: rethinking construct proliferation through meaning, practice, and taxonomy

**DOI:** 10.3389/fpsyg.2026.1871558

**Published:** 2026-07-01

**Authors:** Lorne M. Hartman

**Affiliations:** Translational Research Program, Temerty Faculty of Medicine, University of Toronto, Toronto, ON, Canada

**Keywords:** academic-practice gap, construct proliferation, jingle-jangle fallacy, semantic tools, taxonomic incommensurability

## Abstract

Construct proliferation (CP) in organizational research is typically framed as a problem of redundancy, multiple constructs that differ in name but not in substance. While this framing captures an important aspect of CP, it is incomplete. In this Perspective, I argue that prevailing approaches overemphasize the jangle side of the jingle–jangle fallacy while neglecting the equally consequential problem of semantic divergence under shared labels. I further propose that CP reflects deeper structural dynamics, including misalignment between academic and practitioner audiences and a lack of coordination in how constructs are defined, used, and evaluated. These dynamics contribute to what can be understood as taxonomic incommensurability, where constructs cannot be meaningfully integrated despite apparent similarity. Finally, I outline how recent advances in semantic analysis offer a promising, underutilized tool for addressing CP at the level of meaning rather than measurement alone. Addressing CP, therefore, requires not only improved psychometric practices but a broader shift toward conceptual clarification, cross-domain coordination, and stronger integration between research and application.

## Introduction

Construct proliferation (CP) has become a persistent concern in organizational psychology and management research. The dominant framing characterizes CP as a problem of redundancy: the introduction of new constructs that overlap substantially with existing ones while offering limited incremental explanatory value (Cronbach and Meehl, 1955). This perspective has led to important calls for greater conceptual discipline, improved construct validation, and restraint in introducing new labels (Bowling et al., 2026).

However, despite decades of recognition, CP persists as a systemic issue within organizational science (Murphy and Aguinis, 2018; Spector et al., 2014). This suggests that prevailing explanations may be incomplete. If CP were primarily a technical problem of insufficient psychometric rigor, existing methodological tools would be more effective in constraining it. Instead, proliferation continues even in areas with sophisticated measurement practices (Shaffer et al., 2015).

This Perspective argues that CP reflects not only redundancy but also deeper tensions concerning meaning, audience, and construct purpose. Three issues are underemphasized in current treatments: (1) jingle–jangle asymmetry, (2) the academic–practice gap, and (3) taxonomic incommensurability.

Although the present discussion focuses on organizational psychology, the issues examined are not unique to organizational science. Similar concerns regarding construct proliferation, conceptual overlap, and fragmented taxonomies have been documented across numerous areas of psychology and related disciplines. Organizational psychology serves as a particularly useful illustrative context because of its extensive history of construct development and ongoing debates regarding conceptual redundancy. Nevertheless, the mechanisms discussed here, including taxonomic incommensurability, communication failures, and the separation of academic and practitioner communities, likely operate in many scientific domains where complex phenomena resist straightforward classification.

### The jingle–jangle asymmetry

The concepts of jingle and jangle fallacies have deep roots in psychological measurement. Thorndike (1904) introduced the jingle fallacy to describe the mistaken assumption that measures bearing the same name necessarily assess the same underlying construct. Kelley (1927) later identified the converse problem, the jangle fallacy, in which different labels create the appearance of distinct constructs despite substantial conceptual overlap. Together, these fallacies highlight enduring challenges in construct definition and classification. Although recognized for more than a century, the balance between these two forms of error has received uneven attention, with contemporary concerns about construct proliferation focusing primarily on jangle fallacies while giving comparatively less consideration to jingle fallacies.

This asymmetry is understandable, as redundancy is relatively tractable using standard psychometric tools such as factor analysis and tests of discriminant validity (Lambert and Newman, 2022). However, this emphasis neglects the complementary jingle fallacy: the use of similar labels to describe constructs that differ meaningfully in conceptual core, scope, or mechanism. In many contemporary debates, the issue is not that constructs are redundant, but that shared terminology obscures important distinctions (Podsakoff et al., 2016).

This asymmetry has consequences. When CP is framed primarily as redundancy, conceptual diversity is easily misclassified as unnecessary proliferation. This, in turn, incentivizes consolidation rather than clarification, potentially suppressing theoretically meaningful distinctions. Progress, however, depends not on reducing the number of constructs, but on improving the precision with which their meanings and boundaries are specified (Kuhn, 1962). Although the concept of taxonomic incommensurability is related to Kuhn’s (1962) discussion of paradigm incommensurability, the two should not be conflated. Kuhn’s (1962) concern was with communication and comparison across competing paradigms that organize reality according to different conceptual frameworks. The present discussion focuses instead on the challenges that emerge when researchers working within the same general scientific enterprise develop alternative classifications and labels for complex phenomena. In this sense, taxonomic incommensurability concerns competing ways of carving up the same conceptual terrain rather than competition between fundamentally different paradigms.

### Conceptual fragmentation in organizational scholarship

One illustration of the field’s concern with jangle fallacies can be found in the proliferation of constructs describing positive psychological attachment to work. Constructs such as job satisfaction (e.g., Locke, 1976), work engagement (Schaufeli et al., 2002), job involvement (Kanungo, 1982), work passion (Vallerand et al., 2003), and even newer constructs such as thriving at work (Spreitzer et al., 2005) and “love of the job” (Inness et al., 2026) are often presented as conceptually distinct. Yet many operational definitions and measurement items appear strikingly similar. For example, job satisfaction measures commonly include items such as “All in all, I am satisfied with my job” (Judge et al., 1998), whereas work engagement scales include items such as “I am enthusiastic about my job” and “My job inspires me” (Schaufeli et al., 2002). Work passion measures include items such as “My work is a passion for me” (Vallerand et al., 2003), and the “love of one’s job” scale includes “My work is more than a job to me, it is a passion” (Inness et al., 2026). Although each construct emphasizes somewhat different theoretical nuances, all attempt to capture favorable psychological relationships between individuals and their work. Such examples have motivated concerns that organizational scholarship often generates multiple labels for highly overlapping phenomena, thereby increasing conceptual redundancy and contributing to construct proliferation.

Although construct proliferation is mostly associated with jangle fallacies, the converse problem can also occur when broad labels encompass multiple distinct phenomena. The organizational commitment literature illustrates the complexity of this issue, as affective, continuance, and normative commitment represent different forms of attachment despite sharing a common label (Meyer and Allen, 1991). However, subsequent research has documented substantial overlap between affective and normative commitment, raising questions about their distinctiveness (Ko et al., 1997; Meyer et al., 2002; Solinger et al., 2008). Ironically, Meyer et al.'s (2002) own meta-analysis found that affective and normative commitment were strongly correlated (*ρ* ≈ 0.63), whereas continuance commitment showed much weaker relationships with both. Rather than a straightforward jingle fallacy, the three-component model, therefore, reflects an effort to differentiate forms of attachment while simultaneously highlighting the challenges of determining when conceptually related dimensions constitute distinct constructs. The organizational commitment literature thus occupies an intermediate position between jingle and jangle concerns: a common label encompasses multiple forms of attachment, yet some of those forms may themselves be only partially distinct.

Other examples suggest that the potential for jingle fallacies extends beyond organizational commitment. The label “organizational citizenship behavior” (OCB), for example, encompasses a diverse set of discretionary behaviors including helping, voice, civic virtue, conscientiousness, and sportsmanship. Although these behaviors share a common label, helping a coworker with a difficult task, speaking up to challenge organizational practices, and maintaining a positive attitude in the face of inconvenience may reflect quite different psychological processes and may be associated with different antecedents and outcomes (Podsakoff et al., 2000). Similarly, “organizational justice” is often discussed as a unified phenomenon even though distributive justice concerns the fairness of outcomes, procedural justice concerns the fairness of decision-making processes, interpersonal justice concerns the quality of interpersonal treatment, and informational justice concerns the adequacy and honesty of explanations provided (Colquitt, 2001). These dimensions reflect different evaluative judgments despite frequently being discussed under a common label.

The concept of “organizational culture” presents an even greater challenge, as the same label has been used to refer variously to shared values, norms, assumptions, practices, symbols, and climate perceptions, leading some scholars to question whether culture represents a single construct at all (Denison, 1996). Moreover, the boundaries between culture and other organizational constructs may themselves be ambiguous. For example, employees’ perceptions of organizational culture are often shaped by their experiences with leaders, raising the possibility that commonly observed relationships between leadership and culture partly reflect construct overlaps rather than distinct causal processes (Hartman, 2026). Likewise, the term “leadership” has been applied to an extraordinarily broad range of phenomena, including traits, behaviors, relationships, identities, and social influence processes, prompting recurring concerns about conceptual ambiguity and fragmentation (Avolio et al., 2009). In each case, a common label may create the appearance of conceptual unity while encompassing substantively different psychological or organizational phenomena, illustrating the challenges posed by potential jingle fallacies in organizational scholarship.

Taken together, these examples illustrate that construct proliferation can occur in two directions: scholars may assign different labels to highly similar phenomena (jangle fallacies) or apply the same label to substantively different phenomena (jingle fallacies). Both processes can obscure conceptual boundaries and complicate cumulative knowledge development.

### Construct proliferation and the academic–practice gap

A second driver of CP is the growing disconnection between academic theorizing and organizational practice (Bartunek and Rynes, 2014; Hartman, 2025). Constructs often proliferate not because they are empirically indistinguishable, but because they are weakly constrained by real-world application (Christian et al., 2011; Rynes et al., 2001; Shapiro et al., 2007). Academic incentives prioritize novelty, differentiation, and theoretical contribution, whereas practitioners prioritize usability, parsimony, and decision relevance (Aguinis et al., 2018; Banks et al., 2016).

When constructs are developed primarily within academic discourse, there is limited pressure to determine whether new labels improve prediction, explanation, or intervention beyond existing frameworks already in use. This misalignment contributes to fragmentation (Cascio and Aguinis, 2008). Constructs may be internally coherent within specific research streams yet fail to stabilize across communities of use. As a result, proliferation reflects not only overlap among constructs, but a lack of coordination about what constructs are for and how they should function across contexts (Bartunek and Rynes, 2014).

### From proliferation to taxonomic incommensurability: the promise of semantic approaches

These issues converge in a more fundamental challenge: taxonomic incommensurability (Wulff and Mata, 2025). Taxonomic incommensurability refers to the absence of a coherent classification system linking constructs, definitions, and measures, making it difficult to determine whether constructs represent the same phenomenon, distinct phenomena, or partially overlapping phenomena. When constructs differ in meaning despite similar labels, or share meaning despite different labels, comparisons across studies become difficult, even when empirical data are available.

Taxonomic incommensurability differs from redundancy, overlap, and discriminant validity. Whereas those concepts concern empirical or conceptual similarity between constructs, taxonomic incommensurability concerns the absence of a framework for interpreting such similarities. Traditional psychometric methods are therefore better suited to detecting redundancy than resolving meaning mismatches.

Reframing CP as a problem of taxonomy highlights the need for tools that operate at the level of conceptual representation. Without shared reference points for what constructs mean, cumulative knowledge remains difficult to achieve, regardless of measurement sophistication. Recent advances in natural language processing offer a promising complement to traditional approaches. By embedding construct labels, scale items, and theoretical descriptions into shared semantic spaces, these methods enable the quantification of similarity and divergence at the level of meaning. Early applications (Devlin et al., 2019; Fyffe et al., 2023; Grand et al., 2022; Speer et al., 2022; Wulff and Mata, 2025) suggest that semantic tools can:

- Identify latent redundancies across constructs
- Detect cases where identical labels mask different conceptual content
- Reveal misalignment between theoretical definitions and measurement instruments

When combined with approaches such as prototype analysis (Petersen et al., 2025), semantic methods provide a way to examine how constructs are understood and used across contexts. This opens the possibility of addressing CP not only through consolidation but through systematic clarification and alignment.

Semantic methods identify patterns in language use rather than conceptual identity itself. Consequently, they should be viewed as tools for generating hypotheses about construct similarity rather than definitive tests of redundancy or distinctiveness.

### Bridging psychometric and semantic approaches to construct proliferation

To illustrate the distinction between traditional psychometric approaches and emerging semantic methods, Table 1 contrasts how each framework conceptualizes and diagnoses construct proliferation. Rather than treating these approaches as substitutes, the comparison highlights their complementary strengths: psychometric methods operate on observed covariation, whereas semantic approaches operate on meaning. Together, they offer a more complete toolkit for addressing both redundancy and taxonomic incommensurability.

**Table 1:** Contrasting psychometric and semantic approaches to construct proliferation.

Dimension	Psychometric approach (jangle focus)	Semantic embedding approach (meaning/taxonomy focus)
Primary problem framing	Construct proliferation as redundancy (different labels, same underlying construct)	Construct proliferation as taxonomic incommensurability (misalignment between labels, items, and meanings)
Core fallacy addressed	Jangle fallacy	Jingle–jangle asymmetry (simultaneously detects both)
Level of analysis	Observed data (item responses, scale scores)	Semantic content (labels, items, construct definitions)
Primary data source	Survey responses	Text (scale items, construct labels, theoretical descriptions)
Key analytical tools	Factor analysis, correlations, discriminant validity tests	Sentence embeddings, cosine similarity, clustering algorithms
Construct similarity assessment	Empirical correlations between scales	Cosine similarity between embedded representations of items, scales, and labels
Internal consistency (reliability)	Directly estimated (e.g., Cronbach’s alpha)	Predicted via within-scale semantic similarity and Spearman–Brown formula
Structural fidelity	Factor loadings and model fit indices	*Z*-score comparing within-scale vs. between-scale item similarity
Item–scale relations	Item-total correlations from observed data	Predicted item–scale correlations from embeddings (validated against empirical correlations)
Label–scale alignment	Assumed but rarely tested	Explicitly quantified via cosine similarity between label and scale embeddings
Detection of jangle fallacy	High empirical correlations between differently named constructs	High semantic similarity between scales despite different labels
Detection of jingle fallacy	Difficult to detect	High similarity in labels with low similarity in underlying scale embeddings
Heuristic classification criteria	Based on statistical thresholds (e.g., AVE, HTMT ratios)	Based on similarity distributions (e.g., lower 80% = distinct; upper 20% = overlapping constructs)
Clustering and taxonomy	*Post hoc* factor structures	Unsupervised clustering of scales based on semantic similarity (revealing latent taxonomies)
Strengths	Strong for detecting empirical redundancy and validating measurement structure	Strong for diagnosing meaning-based misalignment and uncovering hidden structure across constructs
Limitations	Limited sensitivity to semantic differences; cannot detect mismatches in meaning	Dependent on quality of text representations; requires validation against empirical data
Implication for CP	Encourages construct consolidation	Encourages construct clarification, alignment, and coordinated taxonomy development

This perspective does not seek to extend Wulff and Mata's (2025) concept of taxonomic incommensurability. Rather, it examines how their account complements Bowling et al.'s (2026) communication-based explanation of construct proliferation.

The argument advanced here is that construct proliferation is best understood as a “both–and” phenomenon. Some proliferation reflects genuine taxonomic challenges arising from the multidimensional and evolving nature of psychological phenomena, whereas other instances reflect failures of cumulative scholarship, communication, and construct integration. Understanding how these processes interact provides a more comprehensive account of why construct proliferation persists despite widespread recognition of its costs. These approaches address different layers of the same problem: psychometric methods evaluate how constructs behave empirically, whereas semantic approaches evaluate what constructs mean.

### Implications for organizational research and practice

Addressing CP requires a shift in how constructs are conceptualized and evaluated. Rather than treating constructs as discrete theoretical contributions, they can be understood as shared tools whose value depends on their ability to organize experience, support inference, and guide action. This shift has several implications:

- Research: greater emphasis on conceptual clarity and boundary specification.
- Practice: greater attention to usability and decision relevance.
- Field: recognition that CP reflects structural and epistemic dynamics as well as methodological issues.

## Conclusion

Construct proliferation is not simply a problem of too many names for the same phenomenon. It reflects deeper tensions concerning meaning, audience, and the role of constructs in organizational science. A narrow focus on redundancy addresses symptoms while leaving underlying drivers intact.

Addressing CP requires attention to jingle fallacies, the academic–practice gap, and taxonomic incommensurability. Semantic approaches offer a promising complement to traditional psychometric methods by focusing on meaning rather than measurement alone.

## Data Availability

The original contributions presented in the study are included in the article/supplementary material, further inquiries can be directed to the corresponding author.
